# Cerebrovascular mental stress reactivity is impaired in hypertension

**DOI:** 10.1186/1476-7120-7-32

**Published:** 2009-07-03

**Authors:** Tasneem Z Naqvi, Hanh K Hyuhn

**Affiliations:** 1 Division of Cardiology at Cedars Sinai Heart Institute, Cedars-Sinai Medical Center, University of Southern California, Los Angeles, USA; 2 Echocardiography Laboratories, Division of Cardiology, Keck School of Medicine, University of Southern California, Los Angeles, USA

## Abstract

**Background:**

Brachial artery reactivity in response to shear stress is altered in subjects with hypertension. Since endothelial dysfunction is generalized, we hypothesized that carotid artery (CA) reactivity would also be altered in hypertension.

**Purpose:**

To compare (CA endothelium-dependent vasodilation in response to mental stress in normal and hypertensive subjects.

**Methods:**

We evaluated CA reactivity to mental stress in 10 young healthy human volunteers (aged 23 ± 4 years), 20 older healthy volunteers (aged 49 ± 11 years) and in 28 patients with essential hypertension (aged 51 ± 13 years). In 10 healthy volunteers and 12 hypertensive subjects, middle cerebral artery (MCA) PW transcranial Doppler was performed before and 3 minutes after mental stress.

**Results:**

Mental stress by Stroop color word conflict, math or anger recall tests caused CA vasodilation in young healthy subjects (0.61 ± 0.06 to 0.65 ± 0.07 cm, p < 0.05) and in older healthy subjects (0.63 ± 0.06 to 0.66 ± 0.07 cm, p < 0.05), whereas no CA vasodilation occurred in hypertensive subjects (0.69 ± 0.06 to 0.68 ± 0.07 cm; p, NS). CA blood flow in response to mental stress increased in young healthy subjects (419 ± 134 to 541 ± 209 ml, p < 0.01 vs. baseline) and in older healthy subjects (351 ± 114 to 454 ± 136 ml, p < 0.01 vs. baseline) whereas no change in blood flow (444 ± 143 vs. 458 ± 195 ml; p, 0.59) occurred in hypertensive subjects. There was no difference in the CA response to nitroglycerin in healthy and hypertensive subjects. Mental stress caused a significant increase in baseline to peak MCA systolic (84 ± 22 to 95 ± 22 cm/s, p < 0.05), diastolic (42 ± 12 to 49 ± 14 cm/s, p < 0.05) as well as mean (30 ± 13 to 39 ± 13 cm/s, p < 0.05) PW Doppler velocities in normal subjects, whereas no change in systolic (70 ± 18 to 73 ± 22 cm/s, p < 0.05), diastolic (34 ± 14 to 37 ± 14 cm/s, p = ns) or mean velocities (25 ± 9 to 26 ± 9 cm/s, p = ns) occurred in hypertensive subjects, despite a similar increase in heart rate and blood pressure in response to mental stress in both groups.

**Conclusion:**

Mental stress produces CA vasodilation and is accompanied by an increase in CA and MCA blood flow in healthy subjects. This mental stress induced CA vasodilation and flow reserve is attenuated in subjects with hypertension and may reflect cerebral vascular endothelial dysfunction. Assessment of mental stress induced CA reactivity by ultrasound is a novel method for assessing the impact of hypertension on cerebrovascular endothelial function and blood flow reserve.

## Introduction

Nitric oxide (NO) release from arterial endothelium causes vasodilation in response to physical stress [1], mental stress [2,3] and acetylcholine [3] in healthy subjects. Aging [4], hypercholesterolemia [5], hypertension [6,7], smoking [8], diabetes mellitus [9], and hyperhomocysteinemia [10] are associated with an impaired endothelium dependent vasodilation. Brachial artery reactivity (BART) in response to shear stress assesses endothelial function by measuring arterial dilation [11,12] and is abnormal in patients with coronary artery disease [13-15], and its risk factors [4-12]. This test also assesses forearm arteriolar resistance and blood flow reserve by measuring Doppler velocity shifts before and during hyperemia. Carotid circulation undergoes anatomical and physiological changes in the presence of risk factors for atherosclerosis, however endothelial mediated carotid artery reactivity and cerebral blood flow reserve has not been evaluated.

Cerebrovascular endothelial derived NO mediates local increase in cerebral blood flow during increase in cerebral metabolism in health [16] and disease [15]. Carotid artery (CA) intima wall thickness (IMT) is a marker of atherosclerosis [17] and correlates with coronary risk factors [18,19]. Hypertension is associated with accelerated atherosclerosis and increased CA IMT as well as reduced endothelium-dependent vasodilation in peripheral arteries [20]. This endothelial dysfunction may also be manifest in the cerebrovascular bed. Since common CA supplies 80% blood flow to internal CA and only 20% to external CA, and since it is an ideal site to measure atherosclerotic burden by IMT as well as vascular stiffness via CA distensibility, it may be ideally suited to investigate the effect of atherosclerosis on cerebrovascular blood flow reserve and CA endothelium dependent responses.

Cerebral blood flow velocity correlates with cerebral blood flow measurements [21,22]. Transcranial Doppler (TCD) derived velocities correlate with cerebral blood flow as measured by PET scanning in normal patients [23]. In addition TCD allows assessment of changes in blood flow reliably [24]. TCD allows assessment of increased blood flow in response to brain activity [25]. An equal amount of middle cerebral artery (MCA) blood flow increase occurs on the right and left sides in response to a wide variety of mental activities in normal subjects irrespective of left or right-handedness [26].

To investigate appropriate physiologic stimuli for endothelium-mediated CA vasodilation, we examined the effects of mental stress, likely to produce local increased shear stress within CA as well as increased cerebral blood flow. We hypothesized that CA reactivity as well as cerebral blood flow in response to mental stress would be abnormal in hypertension due to endothelial dysfunction.

## Methods

32 healthy subjects and 28 hypertensive subjects participated in the mental stress protocol. 22 healthy subjects were age matched to hypertensives, whereas 10 were young healthy subjects who were studied to evaluate the effects of mental stress tasks in young healthy state. All studies were performed in the morning hours (between 8 and 11 AM) after an overnight fast. Informed consent was obtained and the study protocol was approved by the Institutional Review Board for Human Subjects.

### Study Recruitment and Eligibility

Subjects older than 18 years, right-handed, normotensive or with essential hypertension, in normal sinus rhythm and able to give informed consent were included. Hypertension was determined from history and treatment with antihypertensive medication or presence of systolic blood pressure (BP) of 136–180 mmHg and diastolic BP of 86–100 mm Hg on 3 separate occasions 1 week apart. Subjects with history of hypercholesterolemia (total cholesterol >240 and/or LDL cholesterol >160 mg/dl), coronary artery disease, congestive heart failure, cerebrovascular disease, diabetes mellitus, smoking during the previous 1 year, known systemic disease or malignancy, plaque in the common CA or its branches or IMT > 1.4 mm in the common CA, use of hormone replacement therapy or oral contraceptives within the last 3 months, neurological disease, learning disability, substance abuse, acute or chronic use of sedative/hypnotic medication, and those on a controlled diet were excluded. Vitamin C, vitamin E, folic acid and anithypertensive medications were withheld for 24 hours prior to the study participation. Antihypertensive medications included beta-blockers (n = 4), ACE inhibitors (n = 8), and calcium channel blocker (n = 1). Remaining hypertensive subjects were recently diagnosed and on no treatment. All subjects had to have completed undergraduate education (4 years high school and 4 years college) for eligibility.

### Study Protocol

Demographic information was obtained prior to a 15-minute rest period in a quiet room. Arm blood pressure was measured by an automated machine. Heart rate was measured by EKG leads placed on the chest wall and displayed on the ultrasound system.

#### Ultrasound Studies

Ultrasound studies were performed using ATL 5000 ultrasound system equipped with a linear array 5–12 MHz variable frequency scan head as well as a 2.5 MHz transducer. The depth of field, gain, transmit focus and resolution mode settings were adjusted in each patient to give the image of maximum clarity with clear definition of intima-media and kept constant throughout the study. CA imaging was performed in the short axis view to visualize jugular vein stacked on top of common CA and then rotated longitudinally to obtain a straight 5–7 cm segment of the CA below the bulb with clear visualization of IMT of the near and far wall of common CA. Ultrasound probe position was marked on the skin with a marker and position as well as baseline orientation of the probe was mimicked during mental stress. All procedures were recorded on videotape and select frames with pulsed wave (PW) Doppler recordings as well as 2D CA images were digitized and stored in the hard as well as in magneto-optical discs. Analog video output was digitized and digital images were transferred to a dedicated Camtronics Vericis workstation (Camtronics Medical Systems Hartland, WI) to enable measurements of CA diameter and CA IMT [27].

#### Ultrasound Measurements

**Figure 1:**
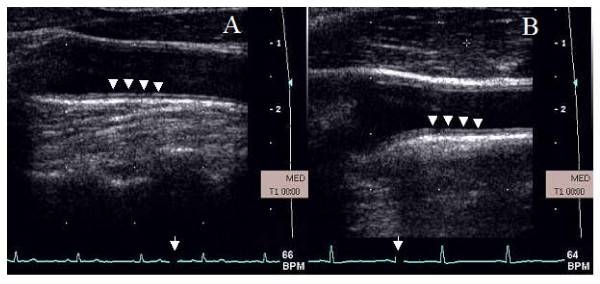
**Carotid artery images in a normotensive (A) and a hypertensive subject (B)**. Intima media thickness is shown by white arrow heads. Carotid intima media thickness was measured in the far wall of the distal right common carotid artery over 1 cm below the carotid bulb. The image was frozen at end diastole (white arrows on EKG strip).

Maximum CA IMT was measured over 1 cm below carotid bulb at the common CA far wall at the onset of QRS complex from intimal-luminal interface to medial adventitial interface. This location was chosen because of its demonstrated reproducibility compared to CA IMT at other sites [28,29]. CA diameter was measured at the onset of QRS complex as the line identifying the media-adventitia interface in the near to the far wall. Figure 1 shows representative CA images from a normal and a hypertensive subject showing intima-media thickness (white arrows). All measurements were made in triplicate and averaged. CA diameter measurements were made over 3 cm of a straight segment below the CA bulb. PW Doppler measurements included: time averaged mean velocity (TAM), peak systolic (PSV), end diastolic velocity (EDV), pulsatility index (PI, (PSV-EDV)/TAM, resistive index (RI, PSV-EDV)/PSV) and blood flow (ml/min = 3.14× (r)^2 ^× TAM × 60). All PW Doppler data was acquired using a 60° angle. CA distensibility was measured at baseline using the formula 2 × % change in CA diameter between systole and diastole/CA diameter systole × pulse pressure [30]. Five averaged Doppler measurements over at least 10 cardiac and 2 respiratory cycles were measured.

#### Protocol: Mental stress

Baseline heart rate and BP were measured. CA ultrasound as described above was performed immediately before after 3 minutes of each of the 3 mental stress tasks. The CA imaging was completed within 60 seconds after stopping each mental stress protocol. We measured serial hemodynamic responses during mental stress and for 10 minutes into recovery and found peak systolic, diastolic and mean BP responses to occur at 3 minutes into mental stress and peak heart rate responses at 4 minutes into mental stress. Thus in the current study, we measured CA reactivity at peak mental stress. A 15-minute inter-stressor interval was allowed for recovery of heart rate and BP as well as CA diameter. Blood flow was displayed on the system as flow = 3.14× (r)^2 ^× TAM × 60, where r = radius of CA. Heart rate was measured continuously and BP pressure every minute during mental stress.

#### Mental Stress Tasks

Three mental stress tasks including 1) Stroop color word conflict test [31], 2) subtraction of serial 7's mathematics test [32] and 3) Ironson anger recall test were assessed in each subject [32]. Tests were performed in a randomized fashion according to the methods previously described [32]. The ultrasound data from the test that produced maximum heart rate and blood pressure response in each subject was selected and is shown in the data.

#### Transcranial Doppler of the Middle Cerebral Artery

Transcranial Doppler (TCD) was performed in a subset of study patients as previously described [33]. A 2.5 MHz transducer was placed in the right temporal region and circle of Willis was identified using cerebral peduncle and posterior communicating artery as landmarks. Depth setting of 14.3 cm was used. MCA was then located by color flow Doppler. Sample volume was placed in the distal 1/3rd of the MCA. Adjustment was made in the position of sample volume using angle correction. PW Doppler measurements PSV, EDV, TAM, RI and PI were performed. With the transducer held in the same location, subject were given instructions for the mental stress task and shown either Stroop or Math slides for 3 minutes as described above depending on which test provided a higher hemodynamic response during CA ultrasound. PW Doppler of the MCA was performed at the end of 3 minutes of mental stress. A 15-minute inter-stressor interval was allowed between CA and MCA ultrasound. Following last mental stress task and 15-minute rest period; 0.4 mg of nitroglycerin (NTG) was administered sublingually. 2D and Doppler CA images were repeated 5 minutes after NTG administration.

#### Timing of Assessment of Vascular Responses

In order to assess the time of peak hemodynamic response, in 8 subjects we examined blood pressure every minute during mental stress and for 10 minutes after mental stress. We found that the maximum hemodynamic response occurs generally within 4 minutes of initiating the stressor, peak responses varying from 30 seconds to 4 minutes into the stressor. These responses declined earlier (within 2 minutes) in normotensives and persisted longer (up to 7 minutes) in hypertensives. The CA flow responses followed this hemodynamic pattern but tended to persist longer (up to 10 minutes) in hypertensive subjects. Given that data acquisition took about 60 seconds, at the end of stressor, we chose to measure CA responses at the end of 3 minutes of mental stress task.

#### Statistical Analysis

Continuous variables are presented as mean ± standard deviation, and categorical variables as counts and percentages. Differences between baseline and peak mental stress variables within each group were compared by Student's paired t-test, between normal (as a single group) and hypertensives by Student's unpaired t-test and between the young normal, old normal and hypertensive groups by ANOVA. A p value of < 0.05 was considered statistically significant.

Relationship of variables such as PSV, TAM, baseline and peak systolic BP, IMT and age was assessed with the dependent variable – delta (absolute and percent) CA diameter, via Pearson correlation coefficient and an F value obtained in the entire study population as well as in hypertensive and the two normal groups. Those variables that were significantly correlated with delta CA diameter (IMT and age) were then used as covariates with baseline systolic BP. Levene's test was used for all variables that correlated with delta CA diameter for homogeneity of variance. Relationships were further confirmed by Dunnett's test and 95% confidence limits derived.

#### Inter-and Intra-observer Variability

All measurements were made in a blinded fashion. For CA diameter, 29 randomly selected images at baseline and peak mental stress were measured by the same observer twice and by 2 observers 1 week apart. Fifteen randomly selected images were used for IMT measurement. Subject variability was assessed by repeating mental stress-induced CA reactivity in 5 subjects 1 week apart. The intra and inter-observer variability was expressed as a percent error for each measurement and were determined as the difference between the 2 observations divided by the mean value of the two observations (×1-×2/(×1+×2)/2*100. Intraobserver variability was 3.4 ± 4% (0.06 ± 0.21 mm), 3.6 ± 4.2% (0.14 ± 0.2 mm) and 2.3 ± 4% (0.09 ± 0.17 mm) for baseline, peak CA diameter measurement and for IMT respectively. Subject variability for CA reactivity was 1.2 ± 2.0% (0.04 ± 0.07 mm). Interobserver variability for CA diameter was 1.1 ± 1.2% (0.01 ± 0.01 mm) and for CA IMT 3.2 ± 2%. These intra-observer and inter-observer variability are comparable to results published earlier [10,34,35].

## Results

### Carotid Artery Reactivity to Mental Stress

We studied 32 healthy human subjects aged 40 ± 15 years, 16 females and 28 hypertensive subjects. 22 healthy volunteers were aged matched to hypertensives and 10 were younger healthy volunteers aged <30 years.

#### Normal Human Volunteers

Two healthy subjects were excluded from analysis, one male subject who did not engage in mental stress tasks and in whom none of the hemodynamic parameters or CA responses changed with mental stress and one female subject in whom mental stress precipitated a transient episode of atrial tachycardia. The female subject subsequently gave a longstanding history of palpitations with mental stress on specific questioning and was subsequently found to have mental stress induced atrial fibrillation. Data on remaining 30 normal subjects (40 ± 15 yrs) is presented. The baseline characteristics of the study population are shown in Table 1. Groups are compared by ANOVA. Amongst 30 normal subjects, there was 1 Asian American, 2 African American women and 2 Persian born American subjects and the remaining 25 subjects were Caucasian American. Among hypertensive subjects, there were 3 Asian American, 2 European born American and the remaining 23 subjects were Caucasian American. There was no difference in education level between noromotensives and hypertensives.

**Table 1:** Baseline Characteristics of the Study Population

Variable	Normotensive (N = 10)	Normotensive (N = 20)	Hypertensive (N = 28)	P-value
Age (year)	23 ± 4	49 ± 11	51 ± 13	0.5126
Male (%)	6 (50)	9 (45)	22 (79)	0.0308
Height (cm)	171 ± 9	171 ± 6.6	173 ± 8.6	0.3256
Weight (kg)	71 ± 16	78.0 ± 17.2	86.1 ± 15.6	0.0947
BMI	25.5 ± 4.19	26.88 ± 5.18	28.27 ± 6.56	0.4356
CA IMT (cm)	0.038 ± 0.01	0.058 ± 0.024	0.070 ± 0.017	0.0448
CA Diameter (cm)	0.61 ± 0.06	0.63 ± 0.06	0.69 ± 0.06	0.0015
Systolic BP(mm Hg)	109 ± 8	118.2 ± 12.5	140 ± 22.2	<.0001
Diastolic BP(mm Hg)	67 ± 7	72.6 ± 9.7	82.2 ± 16.2	0.0137
Mean BP (mm Hg)	81 ± 7	87.6 ± 9.1	100.8 ± 16.5	0.0010
Heart Rate (bpm)	71 ± 12	69.1 ± 12.1	66.4 ± 12.6	0.4689
PSV (cm/s)	105 ± 23	72.2 ± 15.6	74.2 ± 17.9	0.6922
EDV (cm/s)	22 ± 4	19.2 ± 6.0	22.1 ± 5.7	0.0899
TAM (cm)	21 ± 6	18.5 ± 4.9	19.6 ± 5.1	0.4511
RI	0.87 ± 0.1	0.73 ± 0.08	0.81 ± 0.23	0.1341
PI	2.42 ± 0.6	1.73 ± 0.60	1.95 ± 0.96	0.3428
Flow (ml)	381 ± 117	351.4 ± 113.6	444.2 ± 142.9	0.0201
Distensibility dyne-1 cm^2 ^10^-6^	0.84 ± 0.20	0.52 ± 0.26	0.39 ± 0.19	<0.0001

BMI, body mass index; CA, carotid artery; IMT, intima media thickness; BP, blood pressure; PSV, peak systolic velocity; EDV, end diastolic velocity; TAM, time-averaged mean velocity; RI, resistive index; PI, pulsatility index.

Tables 2 and 3 show data on the task that produced the maximum hemodynamic response. Effect of mental stress on CA responses in young and older normal subjects is shown in Table 2 and on older normal and hypertensive subjects is shown in Table 3. Figure 1 shows the effect of mental stress on CA diameter and flow velocities in a normal subject. All subjects achieved an increase in systolic and diastolic BP and heart rate with mental stress except a decrease in mean blood pressure with stress in 3 older healthy subjects.

**Table 2:** Effect of Mental Stress on Carotid Artery Vascular Responses in Young vs Older Normal Subjects

	**Young Normal (n = 10)**		**Older Normal (n = 20)**	
**Variable**	**Baseline**	**Peak**	**Baseline**	**Peak**
Systolic BP (mm Hg)	110 ± 7	124 ± 8*	118 ± 12	131 ± 22*
Diastolic BP (mm Hg)	69 ± 7	82 ± 6*	73 ± 10	79 ± 11*
Mean BP (mm Hg)	83 ± 6	96 ± 7*	88 ± 9	96 ± 11*
Heart Rate (bpm)	71 ± 10	87 ± 13*	69 ± 12	80 ± 17*
CA diameter (cm)	0.61 ± 0.06^†^	0.65 ± 0.07*	0.63 ± 0.06^†^	0.66 ± 0.07*
PSV (cm/s)	110 ± 23	119 ± 28	72 ± 16	76 ± 13
EDV (cm/s)	23 ± 4	25 ± 6	19 ± 6	21 ± 5*
TAM (cm)	22 ± 7	25 ± 6	18 ± 5	23 ± 6*^†^
RI	0.84 ± 0.1	0.79 ± 0.1*	0.73 ± 0.1	0.72 ± 0.1
PI	2.4 ± 0.7	2.1 ± 0.4	1.7 ± 0.6	1.5 ± 0.4
Flow (ml/min)	419 ± 134	541 ± 209*	351 ± 114	454 ± 136*^†^

Values are mean ± SD. Numbers in brackets represent number of participating subjects in each group. Data represents the effects of the mental stress task that produced the maximum increase in blood flow in both groups. *p < 0.05 vs. baseline. ^†^p < 0.05 vs. hypertensive. BP, blood pressure; PSV, peak systolic velocity; EDV, end-diastolic velocity; TAM, time average mean velocity; TAM, time averaged mean velocity. Flow = 3.14× 60× (radius)^2× ^TAM; RI, resistive index; PI, pulsatility index.

**Table 3:** Effect of Mental Stress on Carotid Artery Vascular Response

*Variable*	*Normal (N = 20)*			*Hypertensive (N = 28)*			
	Baseline	Peak	*P-value*	Baseline	Peak	*P-value*	*P-value Normo v. Hyper*
Systolic BP	118.2 ± 12.5	131.3 ± 22.1	0.0035	140.0 ± 22.2	155.3 ± 29.2	<.0001	0.5849
Diastolic BP	72.6 ± 9.7	78.5 ± 10.6	0.0029	82.2 ± 16.2	92.1 ± 18.2	<.0001	0.1241
Mean BP	87.6 ± 9.1	96.2 ± 11.4	0.0003	100.8 ± 16.5	113.7 ± 19.9	<.0001	0.2477
Heart Rate	69.1 ± 12.1	80.1 ± 16.6	<.0001	66.4 ± 12.6	77.4 ± 14.4	<.0001	0.9933
CA Diam	0.63 ± 0.06	0.66 ± 0.07	0.0001	0.69 ± 0.06	0.68 ± 0.07	0.0633	<.0001
PSV	72.2 ± 15.6	75.8 ± 13.2	0.1196	74.2 ± 17.9	70.2 ± 17.4	0.162	0.0495
EDV	19.2 ± 6.0	21.2 ± 5.0	0.0341	22.1 ± 5.7	22.0 ± 8.2	0.5868	0.0841
TAM	18.5 ± 4.9	22.7 ± 6.2	0.0008	19.6 ± 5.1	20.6 ± 8.8	0.4529	0.0764
RI	0.73 ± 0.08	0.72 ± 0.08	0.113	0.81 ± 0.23	0.74 ± 0.18	0.1574	0.338
PI	1.73 ± 0.60	1.51 ± 0.41	0.0581	1.95 ± 0.96	1.61 ± 0.81	0.0471	0.8033
Flow	351.4 ± 113.6	453.6 ± 136.2	<.0001	444.2 ± 142.9	458.1 ± 194.8	0.5851	0.0136

Values are mean ± SD. Numbers in brackets represent number of participating subjects in each group. Data represents the effects of the mental stress task that produced the maximum increase in blood flow in the three groups. PSV, peak systolic velocity; EDV, end-diastolic velocity; TAM, time average mean velocity; RI, resistive index; PI, pulsatility index; TAM, time averaged mean velocity. Flow = 3.14× 60× (radius)^2× ^TAM. Units same as in Tables 1 and 2.

All 3 mental stress tasks caused a mean increase in CA diameter. An increase in CA diameter occurred in 25 healthy subjects (83%), no change from baseline in 4 (2 young and 2 older healthy subjects) and a decrease in one subject (older healthy subject). Twenty-three of 30 (76%) healthy subjects responded with vasodilation. Mean percent change in CA diameter was 5.6 ± 3% in young normal, 4.7 ± 4% in older normal and -2.2 ± 6% in hypertensive subjects (p < 0.0001 vs. all normal subjects). This was associated with a net decrease in resistance and a net increase in flow (delta increase in flow = 109 ± 115 ml) in normal subjects during mental stress.

#### Hypertensive Patients

**Figure 2:**
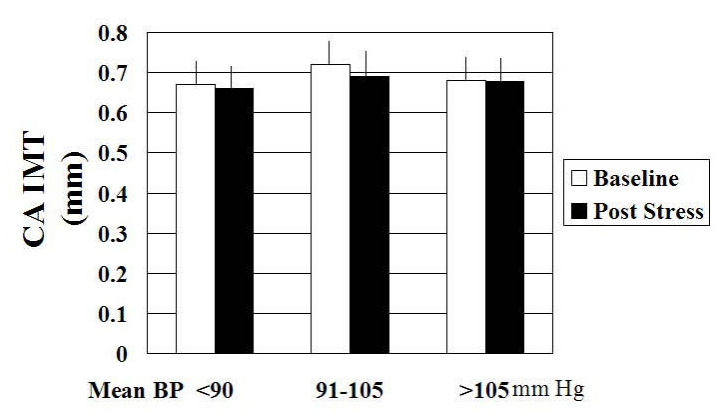
**Bar graphs showing the effect of mental stress on carotid artery diameter in hypertensive subjects with increasing mean blood pressure**.

**Figure 3:**
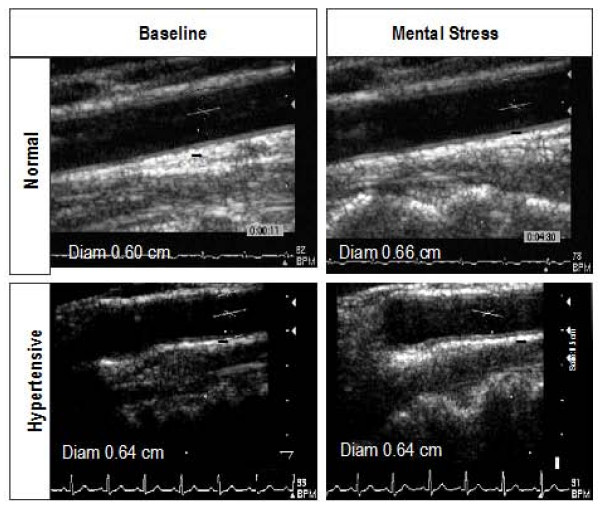
**Representative examples of carotid artery diameter in a normal and a hypertensive subject**. Carotid artery diameter increased after mental stress compared to baseline in the normal subject (top panels) and decreased in response to mental stress compared to baseline in the hypertensive subject (bottom panels). Carotid artery diameter was an average of 10 measurements at the onset of QRS complex.

**Figure 4:**
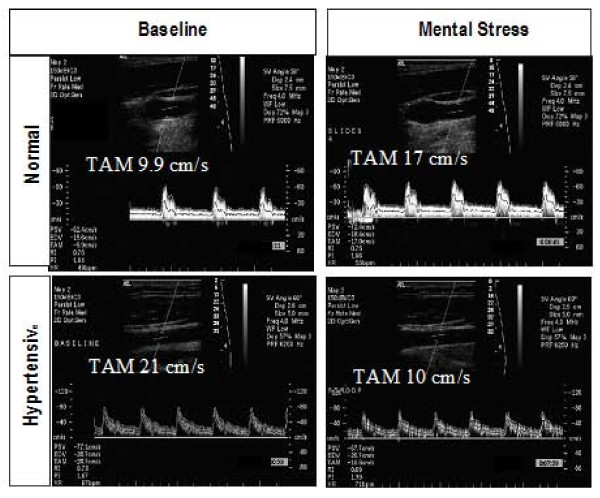
**Representative examples of carotid artery PW Doppler velocity responses to mental stress in a normal and a hypertensive subject**. Carotid artery velocities increased after mental stress compared to baseline in the normal subject (top panels) and decreased in response to mental stress compared to baseline in the hypertensive subject (bottom panels). PSV, peak systolic velocity; EDV, end diastolic velocity; TAM, time averaged mean velocity; RI, resistive index; P, pulsatility index.

We studied 28 hypertensive subjects. Baseline demographic characteristics along with differences with the normal subjects are summarized in Table 1. Hypertensives had a significantly increased size of CA (0.69 ± 0.05 vs. 0.61 ± 0.07 cm, p = 0.003), a significantly higher maximum CA IMT (0.070 ± 0.02 vs. 0.046 ± 0.02 cm, p = 0.005) and decreased CA distensibility (0.39 ± 0.19 vs. 0.62 ± 0.28, p < 0.01) than all normal subjects. The effects of mental stress on CA responses in hypertensive subjects are shown in Table 3. Compared to healthy volunteers, the hypertensive subjects demonstrated significantly reduced CA vasodilation (-2% ± 6% vs. 5 ± 4%, p < 0.0001), and CA flow reserve (3 ± 29 vs. 32 ± 35%, p < 0.01) in response to mental stress. A decrease in CA diameter occurred in 16 hypertensive subjects (52%), no change in 4 and an increase in CA diameter occurred in only 8 hypertensive subjects (29%). No difference in CA vasodilation was observed when hypertensives were divided into three groups based on baseline mean BP of <90, 91–105 and >105 mm Hg as shown in Figure 2. Figure 3 is a representative example of the effect of mental stress on CA diameter in a normal and hypertensive subject and Figure 4 shows effect of mental stress on CA PW Doppler velocities in a normal and a hypertensive subject. No difference in blood pressure response was seen in subjects with treated hypertension in whom medication was held for 24 hours prior to the study vs. untreated hypertensives (data not shown).

We evaluated the carotid % vasodilation in the 3 groups using ANOVA as well evaluated group difference in CA vasodilation after adjusting for baseline IMT and baseline systolic BP. The relationship of baseline systolic BP and percent delta CA diameter had F value of 13.83 (p < 0.0001, Welch's ANOVA F = 14.29, p < 0.0001. To evaluate within individual group differences Dunnett's test was used and showed difference in CA vasodilation in hypertensive vs. young normal (F = 20.27, p < 0.0001, 95% CI for CA vasodilation 3.2–10.5%) as well as hypertensive vs. older normal (F = 16.47, p = 0.0002, 95% CI for CA vasodilation 3.1–12.3%.). Using IMT as a covariate, CA vaodilation in response to mental stress remained significantly different among groups (F for CA vasodilation 12.49, p < 0.001 and F value for IMT 0.84 p = 0.36). Significant group differences also persisted after adjusting for baseline systolic BP (F for CA vasodilation 6.92, p = 0.002 and F value for SBP 0.32, p = 0.57).

In hypertensive subjects, the correlation of mean baseline diastolic BP with baseline CA diameter was 0.37, p = 0.04, and with baseline CA distensibility was -0.49, p = 0.01. We found no significant difference in the change in systolic, diastolic and mean BP in response to mental stress in the 3 groups. However, when we combined the hemodynamic data for all tasks for both groups, a larger increase in systolic BP was seen in the hypertensives vs. normal subjects (27 ± 22 vs.15.5 ± 13 mmHg, p = 0.002), without significant differences in the diastolic (13.6 ± 7 vs. 11 ± 6.5 mmHg, p = 0.17) and mean BP (16 ± 12 vs. 13 ± 7 mmHg, p = 0.16).

### Effects of Individual Tasks

All tasks produced a hemodynamic response. A more pronounced increase in systolic BP occurred in response to math task in hypertensives compared to normals. Changes in CA blood flow and diameter were less pronounced for anger recall task in hypertensive subjects.

### Carotid Artery Reactivity to Nitroglycerin

Nitroglycerin (NTG) responses were assessed in 8 normal and 10 hypertensive subjects. Increase in CA diameter in response to NTG was 5 ± 3% (0.67 ± 0.03 to 0.70 ± 0.04 cm, p < 0.01) in normal and 7 ± 5% (0.72 ± 0.10 to 0.76 ± 0.08 cm, p < 0.01) in hypertensive subjects. NTG decreased mean BP by 3 ± 4% in normals and 5 ± 4% in hypertensives.

**Figure 5:**
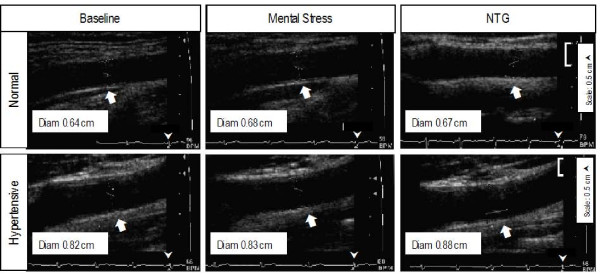
**Representative examples of carotid artery diameter in a normal and a hypertensive subject in response to nigtroglycerin**. Carotid artery diameter increased after mental stress as well as after nitroglycerin compared to baseline in the normal subject (top panels). In the hypertensive subject carotid artery diameter showed no change in response to mental stress but increased after administration of nitroglycerin (bottom panels). Carotid artery diameter was an average of 10 measurements at the onset of QRS complex.

**Figure 6:**
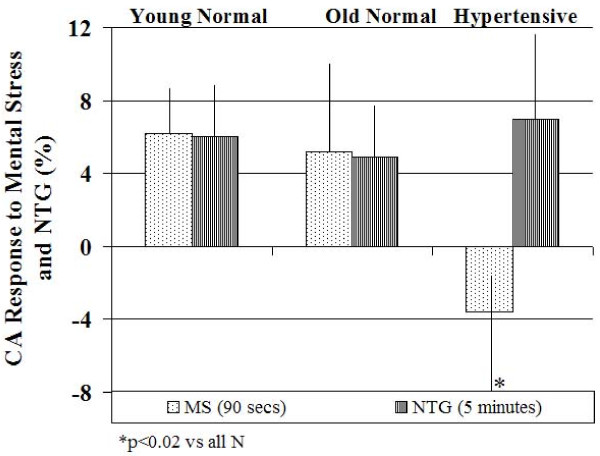
**Comparison of percent change in carotid artery diameter in response to mental stress and nitroglycerin in young normal subjects (n = 4), old normal subjects (n = 4) and hypertensive subjects (n = 10)**. MS, mental stress; N, normal; NTG, nitroglycerin.

Figure 5 shows an example of response to NTG in a normal and a hypertensive subject and Figure 6 compares % CA vasodilation in response to mental stress vs. NTG in normal and hypertensive subjects.

### Blood Pressure and Carotid Artery Reactivity

Correlation of CA diameter during mental stress with blood pressure, age, IMT, distensibility and with PW Doppler variables in normal and hypertensives is shown in Table 4.

**Table 4:** Correlation between Carotid Artery Diameter During Mental Stress and Other Variables in Normal and Hypertensive Subjects

**Variables**	**Normal (n = 30)**	**HTN (n = 28)**
**Blood Pressure Variables**		
SBP_b_	-0.18 (0.35))	-0.05 (0.79)
SBP_p_	-0.21 (0.26)	-0.14 (0.49)
DBP_b_	0.07 (0.7)	0.04 (0.82)
DBP_p_	0.14 (0.45)	-0.10 (0.62)
PSV_b_	0.09 (0.65)	-0.19 (0.33)
PSV_p_	0.04 (0.84)	-0.18 (0.38)
EDV_b_	0.07 (0.72)	-0.40 (0.03)
EDV_p_	0.14 (0.45)	-0.08 (0.68)
TAM_b_	-0.03 (0.87)	-0.41 (0.03)
TAM_p_	-0.09 (0.61)	-0.08 (0.69)
		
**Other Variables**		
Age	-0.24 (0.21)	-0.13 (0.49)
IMT	0.09 (0.64)	0.15 (0.44)
CA Distensibility	0.29 (0.18)	0.03 (0.90)

Numbers represent result of Pearson correlation analysis between % change in carotid artery diameter between baseline and peak mental stress to the listed variables. Numbers in bracket represent the P values. Abbreviations: b, baseline; p, peak stress; SBP, systolic blood pressure; DBP, diastolic blood pressure; PSV, peak systolic velocity; EDV, end-diastolic velocity; TAM, time average mean velocity; IMT, intima media thickness.

### Transcranial Doppler of Middle Cerebral Artery

**Figure 7:**
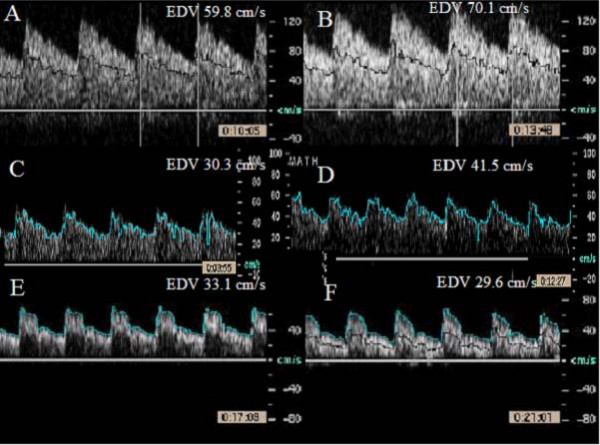
**Effect of mental stress on middle cerebral arterial velocities obtained by transcranial pulsed wave Doppler ultrasound at baseline (left side) and 3 minutes after mental stress (right side)**. Panels A-D are examples from 2 normal subjects and panels E and F from a hypertensive subject.

A subset of study population including 10 healthy subjects (45+13 yrs, 7 F; systolic BP, 118 ± 13 mmHg; diastolic BP, 70 ± 8 mm Hg) and 12 hypertensive subjects (47+8 yrs, 4 F; systolic BP, 130 ± 13 mmHg; diastolic BP, 78 ± 16 mmHg) underwent TCD of MCA. Mental stress induced similar magnitude of increase in heart rate (14 ± 10 vs 15 ± 9%) and mean BP in normal and hypertensive subjects (11 ± 11% vs 9 ± 7%) respectively. MCA flow responses are shown in Table 5 and representative examples of MCA velocities in response to mental stress are shown in Figure 7.

**Table 5:** Effect of Mental Stress on Middle Cerebral Artery Flow Velocities in Normal and Hypertensive Subjects.

	Normal (n = 10)		Hypertension (n = 12)	
MCA Doppler Velocities (cm/sec)	Baseline	Peak	Baseline	Peak
Peak Systolic Velocity	84 ± 22	95 ± 22*	70 ± 18	73 ± 22
End Diastolic Velocity	42 ± 12	49 ± 14*	34 ± 14	37 ± 14
Time Averaged Mean Velocity	30 ± 13	39 ± 13*	25 ± 9	26 ± 9

Values are mean ± SD.*p < 0.05 vs baseline.

## Discussion

The main findings of our study are that in healthy subjects, mental stress causes CA vasodilation. This mental stress-induced CA vasodilation is accompanied by a net increase in CA and MCA blood flow. In hypertensive subjects, mental stress produces no CA vasodilation and no significant change in CA or MCA blood flow. This differential CA response in normal and hypertensive subjects occurs only in response to mental stress and not to the administration of NTG – a direct vasodilator. No significant differences in hemodynamic responses to mental stress were observed in normal and hypertensives. This attenuated CA and blood flow response to mental stress and not to NTG in HTN suggests impaired endothelial function in HTN.

Mental stress produces vasodilation of forearm vasculature in normal humans. This vasodilation has been previously shown to be NO mediated [2] and not catecholamine mediated [36]. Although the magnitude of vasodilation in response to mental stress we observed in our study was less than has been described with BART in subjects with HTN and other risk factors for coronary artery disease [8-11,37-39], the CA vasodilatory responses we observed are comparable to those observed in response to cold pressor test [35,40] by other investigators. Attenuated vasodilator responses have been also shown in atherosclerotic coronary arteries in response to exercise [41,42] and mental stress [44]. Taken together these findings suggest that the mechanism responsible for an attenuated increase in blood flow CA vasodilation in hypertension in our study is likely endothelial dysfunction leading to a decrease in NO availability and impaired cerebral blood flow reserve.

In spontaneously hypertensive rats, reduced cerebral blood flow has been shown to correlate with carotid IMT that improves with long-term antihypertensive treatment [45]. We found increased IMT in hypertensives, however CA vasodilation responses appeared to be independent of baseline IMT as well as to baseline BP. The explanation behind lack of this correlation may lie in our selection process whereby we excluded patients with hyperlipidemia and carotid plaque. Increased IMT in our study in hypertensives may therefore reflect medial vessel hypertrophy as an adaptive response due to increased local transmural pressure due to hypertension and not "atherosclerosism[46]. That is why group classification rather than IMT appeared to be related to group CA vasodilation differences.

We also found larger CA diameter in hypertensives confirming earlier reports [47,48]. In addition, these vessels had reduced distensibility with an inverse relationship between resting BP and CA distensibility. Further increase in BP in response to mental stress may attenuate vasodilatory capacity even further. Indeed preserved vasodilatory response to NTG in hypertensives may be related to hypotensive effects and in turn improved CA distensibility.

No relation was observed between resting BP and CA vasodilatation reserve, however an inverse relationship between baseline velocities and CA vasodilation in response to mental stress observed only in hypertensives may suggest that maximal cerebral vasodilation is present in the resting state in hypertension to preserve cerebral blood flow. This resting maximum vasodilation may impair stress induced vascular reserve.

Although no previous TCD study has examined differences between normal and hypertensives vascular flow reserve, PET studies have demonstrated diminished cerebral blood flow responses during performance of cognitive tasks in hypertensive subjects [49]. Our findings of lack of change of MCA flow velocities in hypertensive subjects suggests impaired cerebral blood flow reserve in hypertensive subjects.

Use of mental stress to assess CA endothelial function is a novel non-invasive technique of assessment of vascular endothelial function. The potential clinical implications of our method are that it can be used in different subsets of patients with risk factors for coronary artery disease other than hypertension. Our approach is simple, relatively time efficient and is highly informative on vascular reactivity at low cost.

Inappropriate vasoconstriction [50,51], or lack of dilation in response to mental stress in stable coronary heart disease, contributes to the genesis of myocardial ischemia and confers an increased risk in patients with coronary artery disease. Whether mental stress induced cerebral ischemia defines subjects at increased risk of future cerebral events needs to be defined.

## Implications

Non invasive assessment of cerebrovascular endothelial function may allow further exploration of cerebrovascular disease pathophysiology, allowing preclinical disease detection and lay out groundwork for future work on examining effects of duration and treatment of hypertension on flow responses to mentally stressful stimuli. Demonstration of direct pathophysiologic link between hypertension and cerebrovascular flow reserve may also provide greater motivation for physicians and patients to better control hypertension and other cardiovascular risk factors. Whether mental stress-induced "cerebral ischemia" affects stroke risk and even more importantly whether poor cerebrovascular flow reserve influences cognition remains to be seen.

## Limitations

These are preliminary findings in a small group of subjects. Even though medications were stopped for 24 hours prior to the study, we cannot rule out the effect of antihypertensive medication on the vascular responses. We did however observe a similar magnitude of increase in heart rate and BP in our study groups. There was a lack of an operator-independent, automatic method to assess IMT and CA reactivity, however all data was measured post procedure blinded to the mental stress responses or subject category. Hypercholesterolemia was excluded by history. We did not measure lipid profile in our study subjects at the time of the study. We did not perform continuous imaging of the CA during mental stress. This was due to interference from speech mandated by mental stress tasks during CA ultrasound. Our data however supports that CA responses were measured at peak hyemodyamic responses, which occurred at the end of mental stress tasks. Finally we did not measure local NO release or effect of NO inhibitor on CA vasodilation responses. However preserved differential CA vasodilation responses to a direct vasodilator vs. mental stress in hypertensives suggest that vasodilation was endothelial mediated. Finally our results apply to treated and untreated subjects with moderate hypertension at the time of the study.

## Competing interests

The authors declare that they have no competing interests.

## Authors' contributions

TZN conceived and designed the study, performed all ultrasound examinations of carotid arteries and transcranial Doppler, modified the mental stress protocols to suite study needs, assisted with data measurement, collection, interpretation and final analysis and drafted the manuscript to its final version. HKH made substantial contributions running the mental stress protocols, data entry and analysis and helped to draft the manuscript. Both authors have given final approval of the version to be published and take responsibility for the content of the manuscript. Both authors read and approved the final manuscript.

## Authors' informations

TZN is currently the Director of Echocardiography at Los Angeles County-USC Medical Center and University of Southern California University Hospital, Medical Director of Non Invasive Diagnostics at the Cardiovascular and Thoracic Institute and Professor of Medicine at the Keck School of Medicine, University of Southern California. At the time this study was conducted she was the Director of Screening and Prevention Ultrasound and of Interventional Echocardiography at Cedars Sinai Medical Center. She is a member of Vascular Council of the American Society of Echocardiography and Chair of the vascular sessions at the American Society of Echocardiography Annual Scientific Sessions at Washington DC 2009. She is a member of Society for Heart Attack Prevention and Eradication (SHAPE Task Force. She has written many articles on the utility of vascular intima-media thickness in cardiovascular risk assessment. She uses carotid IMT as an imaging tool to screen for the presence of subclinical cardiovascular disease and is currently conducting research studies in this area.

HKH was a college science major at University of California, Los Angeles, at the time this study was conducted.
