# Cerebrovascular mental stress reactivity is impaired in hypertension

**DOI:** 10.1186/1476-7120-7-40

**Published:** 2009-08-25

**Authors:** Tasneem Z Naqvi, Hanh K Hyuhn

**Affiliations:** 1 Division of Cardiology at Cedars Sinai Heart Institute, Cedars-Sinai Medical Center, University of Southern California, Los Angeles, USA; 2 Echocardiography Laboratories, Division of Cardiology, Keck School of Medicine, University of Southern California, Los Angeles, USA

## Correction

After publication of this work [1], we noted that Figure Two Y axis is labeled incorrectly as CA IMT (mm). The correct label is ''CA Diameter (cm)'' (see figure 1).

**Figure 1:**
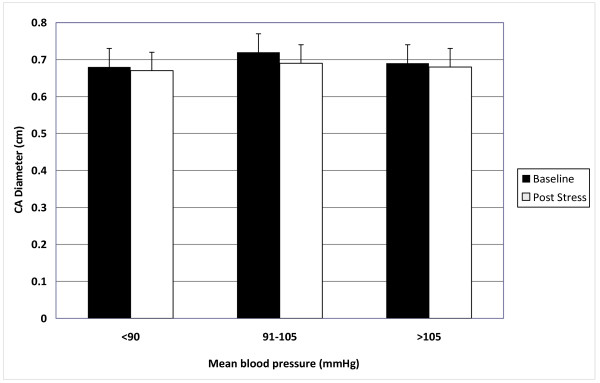
**Figure two - Bar graphs showing the effect of mental stress on carotid artery diameter in hypertensive subjects with increasing mean blood pressure**.

The following statement should also be deleted from the text: "Twenty-three of 30 (76%) healthy subjects responded with vasodilation" under subheading ''Normal Human Volunteers''.
